# Supplementary material for: Involvement of skin TRPV3 in temperature detection regulated by TMEM79 in mice
Source: Nat Commun. 2023 Jul 20;14:4104. doi: 10.1038/s41467-023-39712-x (PMC10359276; doi:10.1038/s41467-023-39712-x)
Supplement: Supplementary file 1 — Supplementary Information [file 41467_2023_39712_MOESM1_ESM.pdf]

Supplementary Data

Involvement of skin TRPV3 in temperature detection regulated by TMEM79 in mice

Jing Lei, Reiko U. Yoshimoto, Takeshi Matsui, Masayuki Amagai, Mizuho A. Kido, and Makoto Tominaga

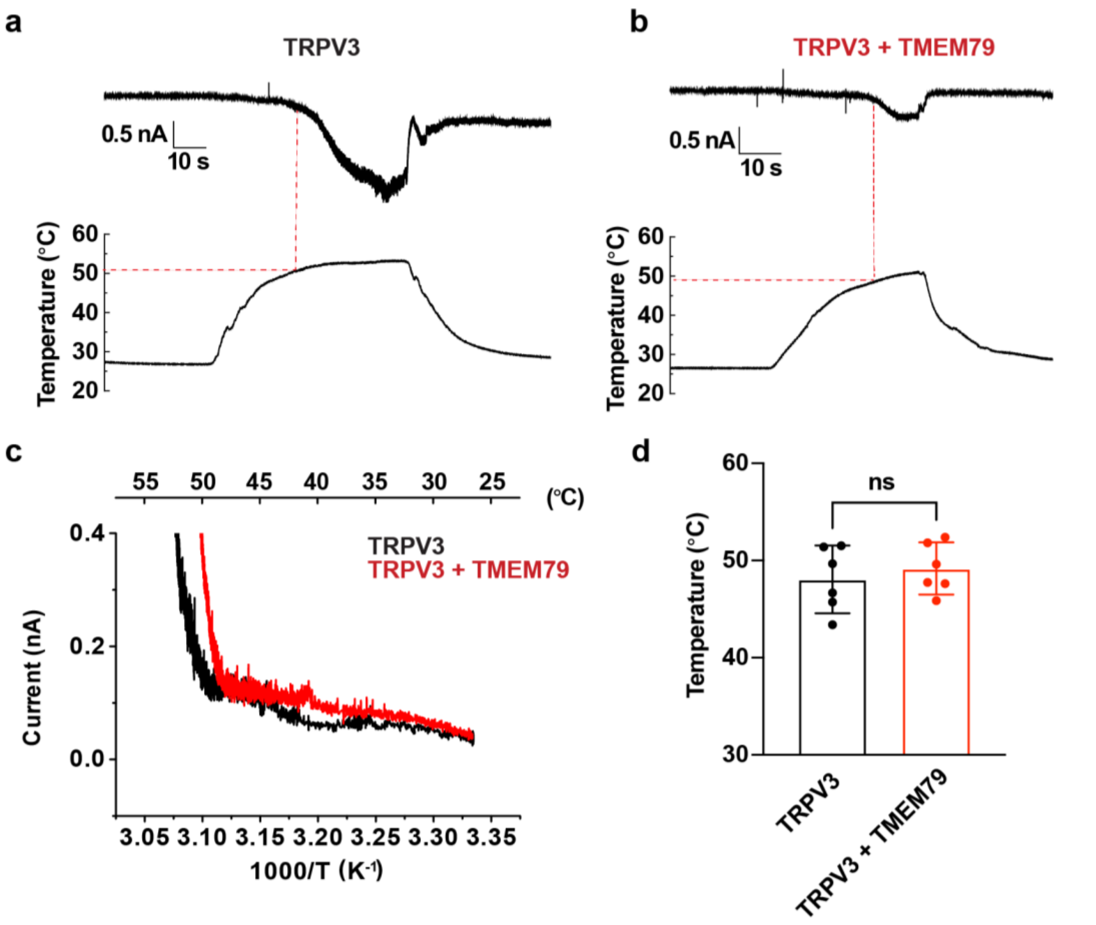

Supplementary Fig. 1: Co-expression of TMEM79 does not affect the temperature thresholds for heat-evoked TRPV3 activation

(a) Representative current and temperature (up to 52°C) traces in HEK293T cells expressing mTRPV3 with a holding potential at -60 mV. (b) Representative current and temperature traces from HEK293T cells co-expressing mTRPV3 and mTMEM79. The red dotted lines indicate the temperature thresholds at which activation starts. (c) Arrhenius plots of TRPV3 currents activated by heat in recordings from (a) and (b). (d) Comparison of TRPV3 temperature thresholds induced by heat in HEK293T cells expressing mTRPV3 and mTRPV3/mTMEM79, n=6 for both. Statistics were performed by a two-tailed unpaired t-test. Error bars indicate SEM for the mean. ns, $P > 0.05$.

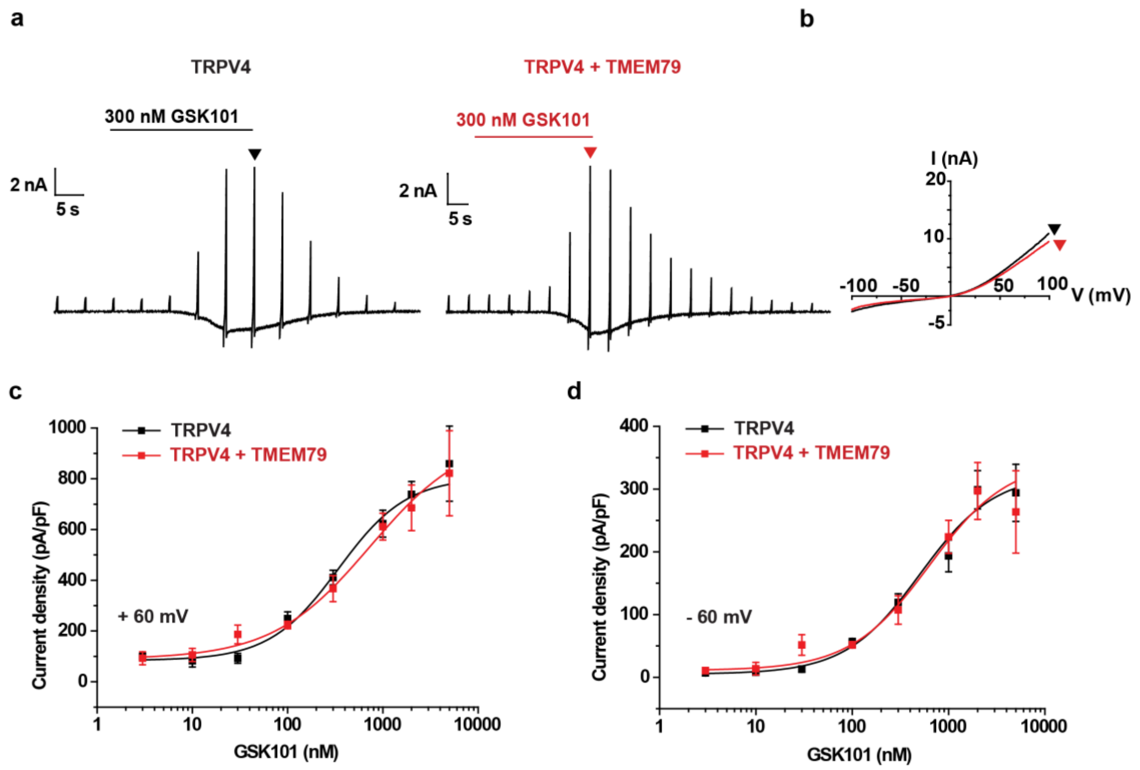

Supplementary Fig. 2: TRPV4-mediated currents are not affected by co-expression of TMEM79

(a) Representative GSK101-evoked (300 nM) current traces in an mTRPV4-expressing HEK293T cell (left) and an mTRPV4/mTMEM79-expressing HEK293T cell (right). Recordings were performed in a ramp-pulse protocol (-100 to +100 mV) every three seconds at a holding potential of -60 mV. Scale bars indicate current amplitudes (y-axis, nA) and time (x-axis, s). (b) I-V curves from the currents are shown in grey, black, and red. (c and d) GSK101 dose-dependent curves in HEK293T cells expressing mTRPV4 alone (black) or co-expressing mTRPV4 and mTMEM79 (red) at +60 mV (c) and -60 mV (d). Current densities (pA/pF) represent the largest values of the GSK101-induced currents. The number of recordings in (c) and (d) is 3, 5, 4, 9, 7, 14, 11 and 11 for cells expressing TRPV4 alone (black) and is 3, 5, 5, 10, 7, 13, 12 and 5 for cells expressing TRPV4 and TMEM79 (red), respectively from low to high concentration of GSK101. All

curves were fitted with the Hill equation with a Hill coefficient (n) around 1. Statistics were performed by a two-tailed unpaired t-test. All error bars and data represent the mean ± SEM.

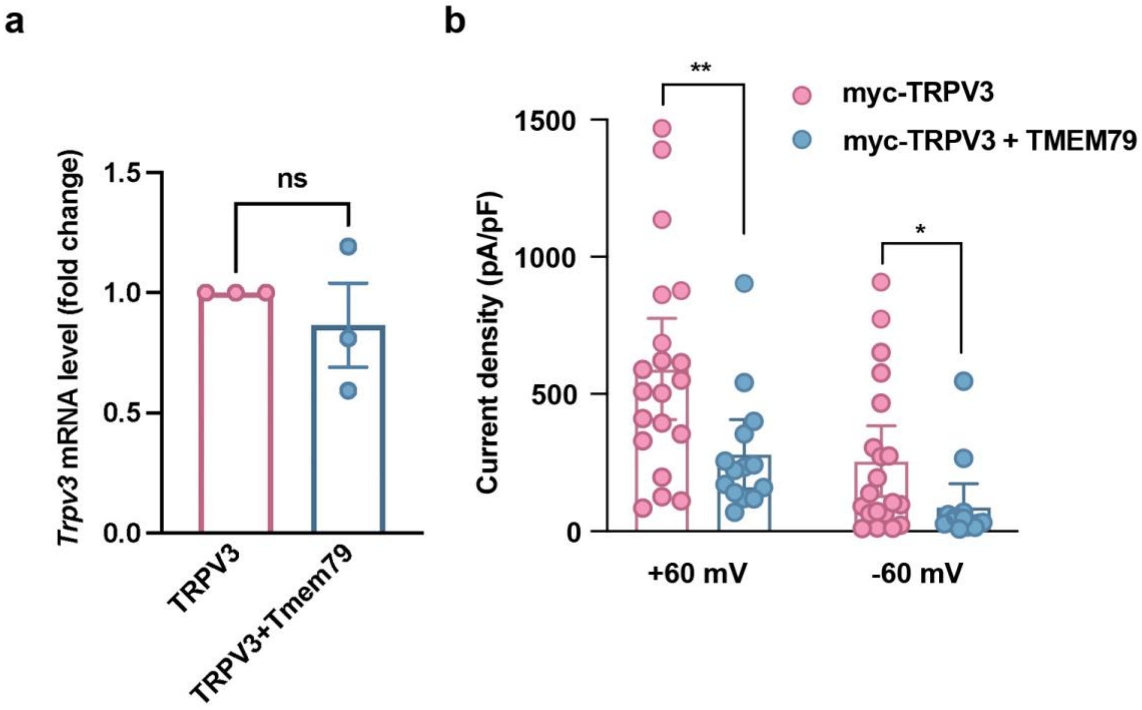

Supplementary Fig. 3: Co-expression of TMEM79 reduces TRPV3-mediated currents without affecting TRPV3 mRNA levels

(a) *Trpv3* mRNA levels in HEK293T cells expressing mTRPV3 or mTRPV3/mTMEM79 (n=3). (b) Comparison of 1 mM 2-APB-induced current densities in HEK293T cells expressing myc-mTRPV3 (red circles, 590.8 ± 88 pA/pF at + 60 mV and 255.3 ± 61.5 pA/pF at - 60 mV, n=20) or expressing both myc-mTRPV3 and mTMEM79 (blue circles, 280 ± 58.7 pA/pF at + 60 mV and 88.2 ± 39.2 pA/pF at -60 mV, n=14) at ± 60 mV. Statistics were performed by a two-tailed unpaired t-test. All data represent the mean ± SEM and all error bars indicate a 95% confidence interval for the mean. *, $P < 0.05$; **, $P < 0.01$; ns, $P > 0.05$.

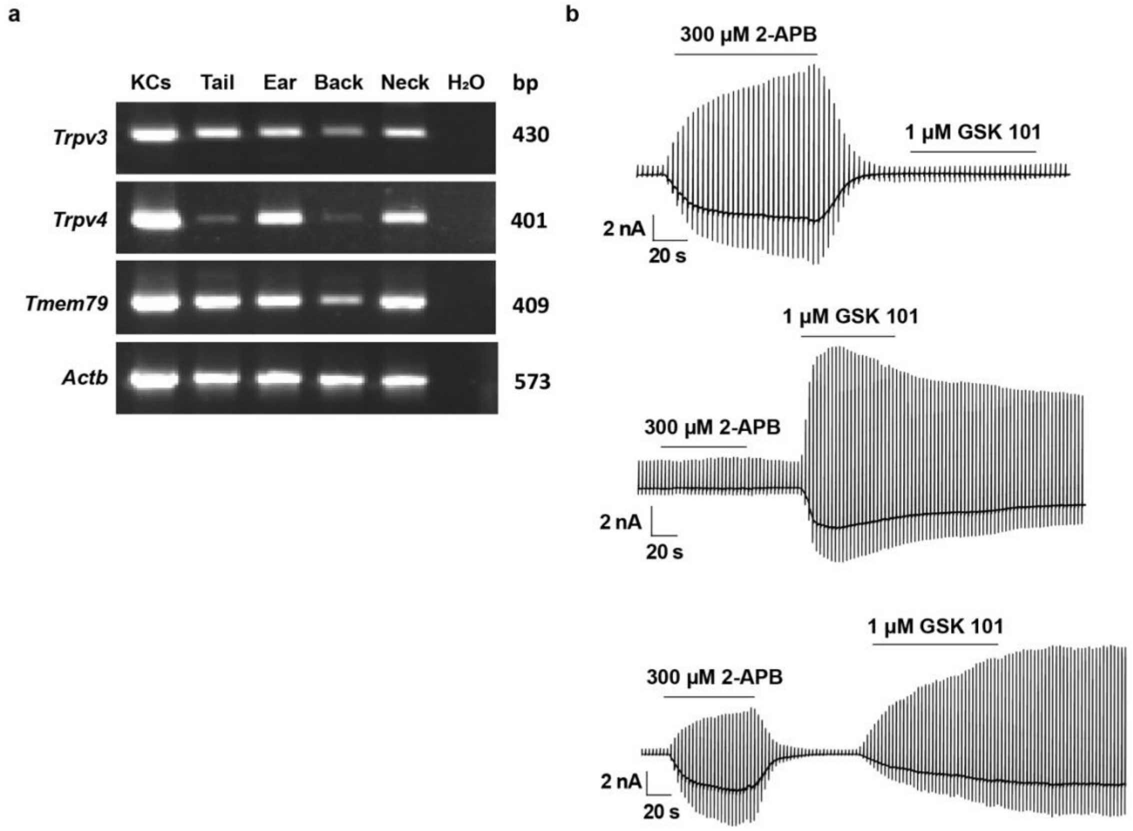

Supplementary Fig. 4: Most mouse keratinocytes functionally express both TRPV3 and TRPV4

(a) Agarose gel electrophoresis of RT-PCR products. mRNA expression of *Trpv3*, *Trpv4*, *Tmem79*, and *Actb* were amplified from tail keratinocytes (KCs), and skin tissues of the tail, ear, back, and neck from a wild-type mouse. (b) 300 μM 2-APB and 1 μM GSK101 induced three types of currents in WT primary keratinocytes. (top) Cells responding to 2-APB alone (12.7%). (middle) Cells responding to GSK101 alone (3.2%). (bottom) Cells responding to both 2-APB and GSK101 (84.1%).

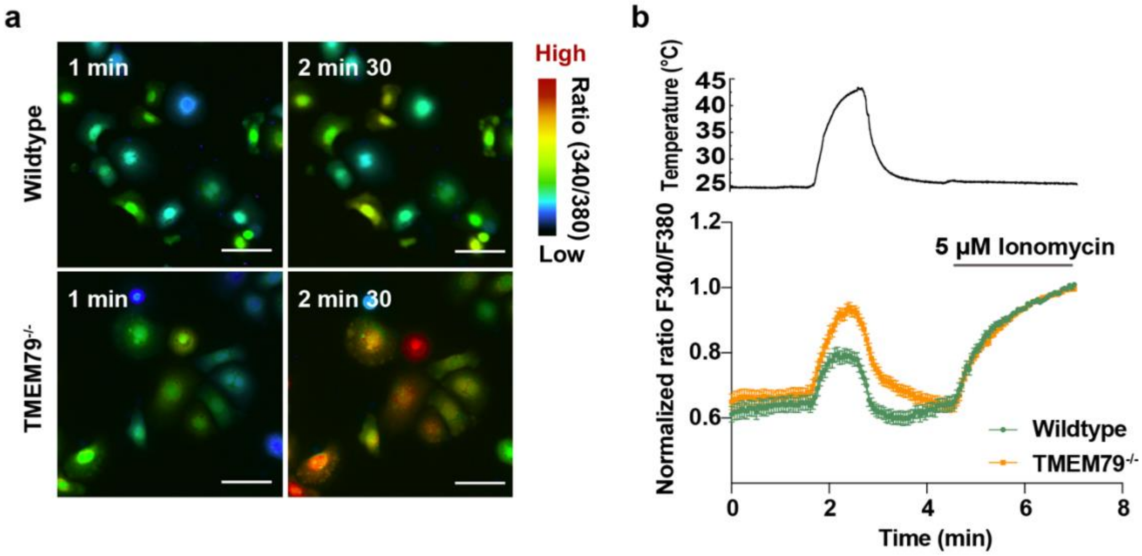

Supplementary Fig. 5: Increase in intracellular Ca^{2+} concentrations induced by heat stimulation in mouse keratinocytes

(a) Representative fluorescence images showing changes in the Fura-2 ratios in primary keratinocytes from wild-type and TMEM79$^{-/-}$ mice in response to heat. Scale bars indicate 50 μm. (b) Changes in normalized (to 5 μM ionomycin-evoked responses) Fura-2 ratios (340/380) induced by heat stimulation in primary keratinocytes derived from wild-type (green) and TMEM79$^{-/-}$ (orange) mice. n = 244 (green) and 303 (orange) cells, respectively from three times repeats. Error bars indicate a 95% confidence interval for the mean.

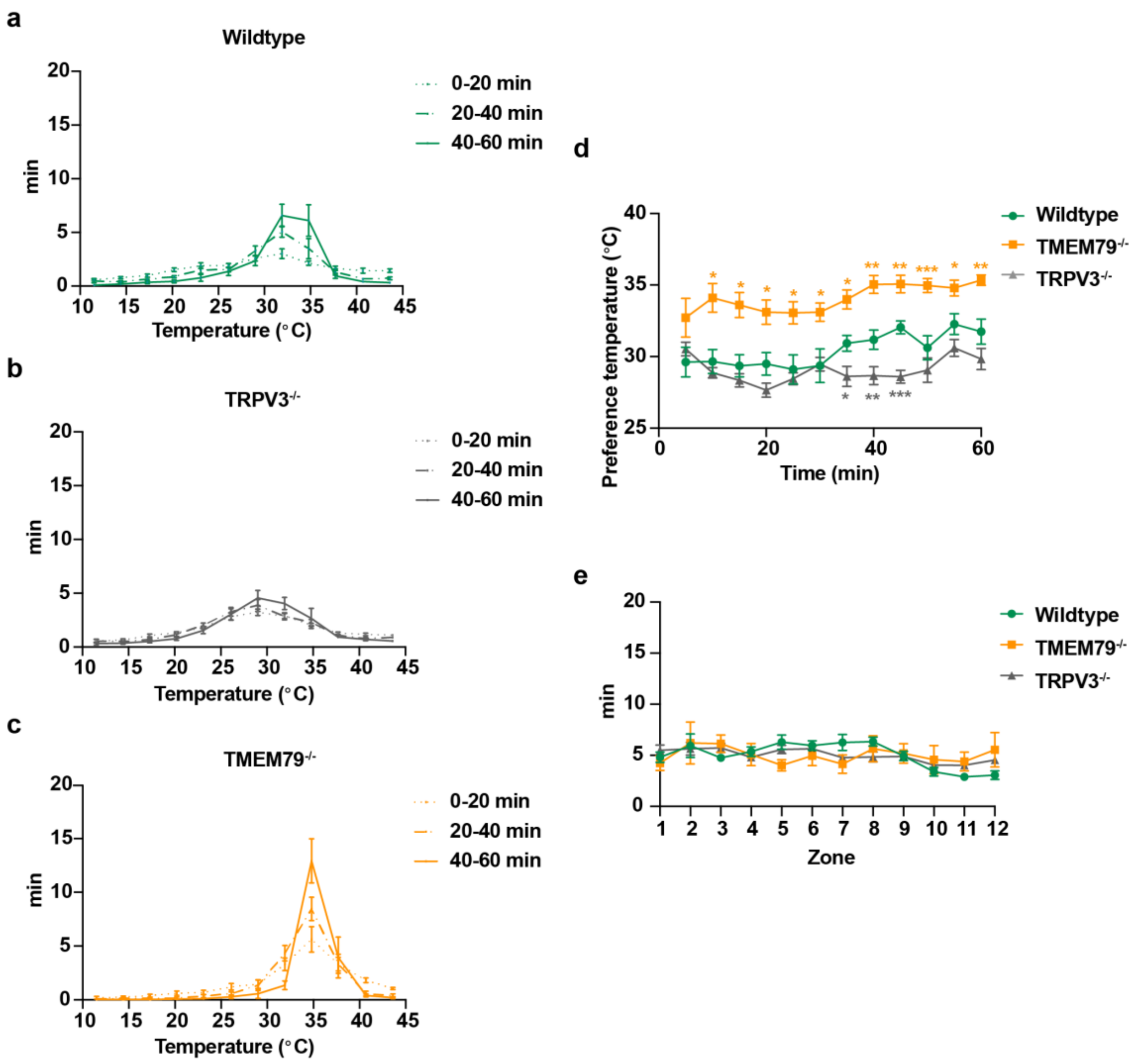

Supplementary Fig. 6: Knockout of TMEM79 affects temperature-dependent behaviors in mice

(a, b, and c) "Spending time" of mice from wild-type (a), *Trpv3*$^{-/-}$ (b), and *Tmem79*$^{-/-}$ (c) genotypes on a thermal gradient ring with a 20-minute interval. (d) The average preferred temperature of mice at each 5-minute interval. Statistics were performed by a mixed-effects (two-way) ANOVA with Geisser-Greenhouse correction. Multiple comparisons were performed without correction. (e) Amount of time the mice spent on a thermal gradient ring without a thermal gradient at room temperature (25°C) during 1 hour of free

movement. In figure (a-d), n = 10, 14, and 10 for wild-type, *Trpv3*$^{-/-}$, and *Tmem79*$^{-/-}$ mice, respectively. In figure (e), n = 7, 5, and 6 for wild-type, *Trpv3*$^{-/-}$, and *Tmem79*$^{-/-}$ mice, respectively. All error bars represent the mean ± SEM. *, P < 0.05; **, P < 0.01; ***, P < 0.001.

Supplementary Table 1

Primer list for genotyping (# 1-3), subcloning (#4-5), RT-PCR (# 6-13), and qPCR (# 14-17).

**Primer order (#)**	**Primer name**	**Sequence (5'-3')**
1	*Tmem79-Intron1-F*	ATCCTCCATTTCGCTCCTCTGC
2	*Lar3-universal-R*	CACAACGGGTTCTTCTGTTAGTCC
3	*Tmem79-Exon2-R*	CTAGGGGCTCTGGTTCAATATC
4	*Tmem79-KpnI-F*	ATAGGTACCATGACAGAACCGGAGACACTG
5	*Tmem79-NotI-R*	AATGCGGCCGCTCAGCCCCATGAGC
6	*Trpv3-Exon15-F*	GGACTGCAGTTCCTATGGCA
7	*Trpv3-Exon17-R*	TGCTGTCCGTCTTATGGGTC
8	*Trpv4-F*	ACAACACCCGAGAGAACACC
9	*Trpv4-R*	CCCAAACTTACGCCACTTGT
10	*Tmem79-Exon1-F*	CCTTTCCCATCGTACTCGGG
11	*Tmem79-Exon3-R*	CGAGCATGGTCAGGGTAGTC
12	*ß-actin-F*	TGTTACCAACTGGGACGACA
13	*ß-actin-R*	AAGGAAGGCTGGAAAAGAGC
14	*Trpv3-Exon2-F*	ACGGTCACCAAGACCTCTC
15	*Trpv3-Exon3-R*	GACTGTTGGGATTGGATGGGG
16	*Gapdh-F*	CATCACTGCCACCCAGAAGACTG
17	*Gapdh-R*	ATGCCAGTGAGCTTCCCGTTCAG
