# Supplementary material for: Involvement of skin TRPV3 in temperature detection regulated by TMEM79 in mice
Source: Nat Commun. 2023 Jul 20;14:4104. doi: 10.1038/s41467-023-39712-x (PMC10359276; doi:10.1038/s41467-023-39712-x)
Supplement: Supplementary file 3 — Reporting Summary [file 41467_2023_39712_MOESM3_ESM.pdf]

nature portfolio

Corresponding author(s): DAPR NCOMMS-22-46564A

Last updated by author(s): Jun 11, 2023

Reporting Summary

Nature Portfolio wishes to improve the reproducibility of the work that we publish. This form provides structure for consistency and transparency in reporting. For further information on Nature Portfolio policies, see our Editorial Policies and the Editorial Policy Checklist.

Statistics

For all statistical analyses, confirm that the following items are present in the figure legend, table legend, main text, or Methods section.

n/a	Confirmed	
☐	☒	The exact sample size (n) for each experimental group/condition, given as a discrete number and unit of measurement
☐	☒	A statement on whether measurements were taken from distinct samples or whether the same sample was measured repeatedly
☐	☒	The statistical test(s) used AND whether they are one- or two-sided<br>*Only common tests should be described solely by name; describe more complex techniques in the Methods section.*
☒	☐	A description of all covariates tested
☐	☒	A description of any assumptions or corrections, such as tests of normality and adjustment for multiple comparisons
☐	☒	A full description of the statistical parameters including central tendency (e.g. means) or other basic estimates (e.g. regression coefficient) AND variation (e.g. standard deviation) or associated estimates of uncertainty (e.g. confidence intervals)
☐	☒	For null hypothesis testing, the test statistic (e.g. F, t, r) with confidence intervals, effect sizes, degrees of freedom and P value noted<br>*Give P values as exact values whenever suitable.*
☒	☐	For Bayesian analysis, information on the choice of priors and Markov chain Monte Carlo settings
☒	☐	For hierarchical and complex designs, identification of the appropriate level for tests and full reporting of outcomes
☒	☐	Estimates of effect sizes (e.g. Cohen's d, Pearson's r), indicating how they were calculated

Our web collection on statistics for biologists contains articles on many of the points above.

Software and code

Policy information about availability of computer code

Data collection	Micropipette puller (Sutter, P-97)<br>Manipulator controller (Sutter, MPC-200)<br>Micromanipulator (Sutter, ROE200)<br>Digitizer (Axon, 1440A)<br>Digital storage oscilloscope (Hitachi, VC-6723)<br>Amplifier (Axon, 200B)<br>NIS-Elements AR (Nikon)<br>Thermal gradient ring (Ugo Basile, 35550)<br>Axio Observer (Zeiss , 7 and Z1)<br>LSM confocal with Airyscan (Zeiss, 800 and 880)

Data analysis	pCLAMP 10 (Axon)<br>Clampfit 11.2 (Molecular Devices)<br>Nikon NIS-Elements AR<br>ANY-maze software (Ugo Basile, 35550)<br>ZEN Blue edition 3.5 software (Carl Zeiss)<br>Graphpad Prism 9.1.2<br>Origin 9.0<br>ImageJ (National Institutes of Health, USA)

For manuscripts utilizing custom algorithms or software that are central to the research but not yet described in published literature, software must be made available to editors and reviewers. We strongly encourage code deposition in a community repository (e.g. GitHub). See the Nature Portfolio guidelines for submitting code & software for further information.

Data

Policy information about availability of data

All manuscripts must include a data availability statement. This statement should provide the following information, where applicable:

- Accession codes, unique identifiers, or web links for publicly available datasets
- A description of any restrictions on data availability
- For clinical datasets or third party data, please ensure that the statement adheres to our policy

All data and materials used in the analysis are available in the manuscript and Supplementary Information. Source data are provided with this paper.

Research involving human participants, their data, or biological material

Policy information about studies with human participants or human data. See also policy information about sex, gender (identity/presentation), and sexual orientation and race, ethnicity and racism.

Reporting on sex and gender	n/a
Reporting on race, ethnicity, or other socially relevant groupings	n/a
Population characteristics	n/a
Recruitment	n/a
Ethics oversight	n/a

Note that full information on the approval of the study protocol must also be provided in the manuscript.

Field-specific reporting

Please select the one below that is the best fit for your research. If you are not sure, read the appropriate sections before making your selection.

☒ Life sciences ☐ Behavioural & social sciences ☐ Ecological, evolutionary & environmental sciences

For a reference copy of the document with all sections, see nature.com/documents/nr-reporting-summary-flat.pdf

Life sciences study design

All studies must disclose on these points even when the disclosure is negative.

Sample size	For whole-cell patch-clamp experiments, the expression level of ion channels (TRPV3 or TRPV4) in each single cell for either overexpression system or native keratinocytes is not identical, which is different from stable cell line with consistent expression of target protein. Therefore, recordings with n>5 are required per group. Other molecular experiments are common techniques used in life science field, we defined the sample size based on others' studies and experience.
Data exclusions	The patch-clamp recordings with cell leakage were excluded from analysis.
Replication	All the data were obtained with at least three biological replicates.
Randomization	For in vivo behavioral experiments, to avoid the effect of age or gender, male mice aged at 7-10 weeks were applied for each genotype.
Blinding	Ideally, blinding is required for most studies. However, it was hardly achived because almost all experiments were done by the same investigator. In terms of the confocal images, samples were prepared by one investigator, and imaging and interpretation were done by different investigators.

Reporting for specific materials, systems and methods

We require information from authors about some types of materials, experimental systems and methods used in many studies. Here, indicate whether each material, system or method listed is relevant to your study. If you are not sure if a list item applies to your research, read the appropriate section before selecting a response.

Materials & experimental systems

n/a	Involved in the study
☐	☒ Antibodies
☐	☒ Eukaryotic cell lines
☒	☐ Palaeontology and archaeology
☐	☒ Animals and other organisms
☒	☐ Clinical data
☒	☐ Dual use research of concern
☒	☐ Plants

Methods

n/a	Involved in the study
☒	☐ ChIP-seq
☒	☐ Flow cytometry
☒	☐ MRI-based neuroimaging

Antibodies

Antibodies used	Western blotting: [1st ab]mouse anti-FLAG (Sigma, F3165, 1:2000), rabbit anti-FLAG (Santa Cruz, sc-807, 1:1000), mouse anti-MYC (MBL, M047-3, 1:1000), and HRP-conjugated anti-GAPDH (Cell Signaling, 3683, 1:1000); [2nd Ab] anti-mouse IgG (Cell Signaling, 7076, 1:10000) and anti-rabbit IgG (Cell Signaling, 7074, 1:10000)<br><br>Immunocytochemistry: [1st Ab] rabbit anti-sodium potassium ATPase (Abcam, ab76020, 1:500), mouse anti-MYC (MBL, 1:200), rabbit anti-FLAG (Santa Cruz, 1:50), mouse anti-LAMP1 (Santa Cruz, sc-20011, 1:100), and mouse anti-calnexin (Santa Cruz, sc-46669, 1:100); [2nd Ab] goat anti-mouse IgG (A-11029, Alexa 488 conjugated, Invitrogen, 1:500), goat anti-mouse IgG (A-11032, Alexa 594 conjugated, Invitrogen, 1:500), goat anti-rabbit IgG (A-11034, Alexa 488 conjugated, Invitrogen, 1:500), goat anti-rabbit IgG (A-11037 and Alexa 594 conjugated, Invitrogen, 1:500).
Validation	For both Western blotting and Immunocytochemistry experiments, the validation of anti-MYC and anti-FLAG antibodies were performed with the HEK293T cells without overexpressing the same tagged proteins. For Organelle antibodies, validation was done without inoculating the 1st antibodies.

Eukaryotic cell lines

Policy information about cell lines and Sex and Gender in Research

Cell line source(s)	HEK293T cells
Authentication	ATCC CRT-3216
Mycoplasma contamination	No contamination
Commonly misidentified lines (See ICLAC register)	*Name any commonly misidentified cell lines used in the study and provide a rationale for their use.*

Animals and other research organisms

Policy information about studies involving animals; ARRIVE guidelines recommended for reporting animal research, and Sex and Gender in Research

Laboratory animals	C57BL/6NCr, male, 7-10 weeks old (behaviral experiments), 4-5 weeks old (keratinocyets preparation)
Wild animals	No wild animals were used in this study.
Reporting on sex	All the animal experiments were applied to only male mice in this study. The comparison between male and female will be interesting for a future investigation.
Field-collected samples	The study does not involve samples collected from the field.
Ethics oversight	All procedures were approved by the Institutional Animal Care and Use Committee of the National Institute of Natural Sciences and carried out according to the National Institutes of Health and National Institute for Physiological Sciences guidelines.

Note that full information on the approval of the study protocol must also be provided in the manuscript.
